# Lineage 1 Porcine Reproductive and Respiratory Syndrome Virus Attenuated Live Vaccine Provides Broad Cross-Protection against Homologous and Heterologous NADC30-Like Virus Challenge in Piglets

**DOI:** 10.3390/vaccines10050752

**Published:** 2022-05-10

**Authors:** Hongliang Zhang, Lirun Xiang, Hu Xu, Chao Li, Yan-Dong Tang, Bangjun Gong, Wenli Zhang, Jing Zhao, Shuaijie Song, Jinmei Peng, Qian Wang, Tongqing An, Xuehui Cai, Zhi-Jun Tian

**Affiliations:** State Key Laboratory of Veterinary Biotechnology, Harbin Veterinary Research Institute, Chinese Academy of Agricultural Sciences, Harbin 150001, China; zhanghongliang01@caas.cn (H.Z.); zhhlxlr@163.com (L.X.); xuhu1995@foxmail.com (H.X.); lichao2459@foxmail.com (C.L.); tangyandong@caas.cn (Y.-D.T.); bangjungong@foxmail.com (B.G.); zwl5561@163.com (W.Z.); zhaojing94vet@163.com (J.Z.); m18304637191@163.com (S.S.); pjm7614@163.com (J.P.); wangqian@caas.cn (Q.W.); antongqing@caas.cn (T.A.); caixuehui139@163.com (X.C.)

**Keywords:** PRRSV, lineage 1, NADC30-like, SD-R, pathogenicity, cross-protection efficacy

## Abstract

Porcine reproductive and respiratory syndrome virus (PRRSV) is an important pathogen that endangers the swine industry worldwide. Recently, lineage 1 PRRSVs, especially NADC30-like PRRSVs, have become the major endemic strains in many pig-breeding countries. Since 2016, NADC30-like PRRSV has become the predominant strain in China. Unfortunately, current commercial vaccines cannot provide sufficient protection against this strain. Here, an attenuated lineage 1 PRRSV strain, named SD-R, was obtained by passaging an NADC30-like PRRSV strain SD in Marc-145 cells for 125 passages. Four-week-old PRRSV-free piglets were vaccinated intramuscularly with 10^5.0^TCID_50_ SD-R and then challenged intramuscularly (2 mL) and intranasally (2 mL) with homologous NADC30-like PRRSV SD (1 × 10^5.0^TCID_50_/_mL_) and heterologous NADC30-like PRRSV HLJWK108-1711 (1 × 10^5.0^TCID_50_/_mL_). The results showed that antibodies against specific PRRSVs in 5 of 5 immunized piglets were positive after a 14-day post-vaccination and did not develop fever or clinical diseases after NADC30-like PRRSV challenges. Additionally, compared with challenge control piglets, vaccinated piglets gained significantly more weight and showed much milder pathological lesions. Furthermore, the viral replication levels of the immunized group were significantly lower than those of the challenge control group. These results demonstrate that lineage 1 PRRSV SD-R is a good candidate for an efficacious vaccine, providing complete clinical protection for piglets against NADC30-like PRRSVs.

## 1. Introduction

Porcine reproductive and respiratory syndrome virus (PRRSV), is an enveloped, positive-sense, single-stranded RNA virus of the family *Arteriviridae* and the genus *Porarterivirus*, and is the aetiological agent of porcine reproductive and respiratory syndrome (PRRS), which causes enormous economic losses to the global swine industry [1]. PRRSVs can be divided into two distinct species, *Betaarterivirus suid 1* (PRRSV-1) and *Betaarterivirus suid 2* (PRRSV-2) (ICTV2021). PRRSV-1 is mainly prevalent in Europe, and PRRSV-2 is prevalent in America and Asia; partial subtypes of both PRRSVs can be found across North America, Europe, and Asia [2,3]. In 2010, a phylogenetic lineage-based PRRSV typing method was proposed. This classification system grouped PRRSV-1 strains into four subtypes (subtype I (Global), subtype I (Russia), subtype II and III) and PRRSV-2 strains into nine lineages (lineage 1-lineage 9) based on phylogenetic relationships in the ORF5 region [3,4]. Although subtype I (Global) of PRRSV-1 has been reported in Asia and America, the other subtypes have not been reported in regions other than Europe [5,6,7,8,9,10]. PRRSV-2 has a high degree of genetic diversity, and the nine lineages can be further divided into several sublineages [4]. The earliest reported lineage was lineage 5, which appeared in the United States, and is mainly distributed in the United States, southern Canada, and parts of China [3]. Then, lineages 8 and 9 were discovered throughout the United States [4]; however, it is puzzling that sublineage 8.7 (HP-PRRSV), which was first reported in China in 2006 with the characteristics of causing high temperatures in infected pigs, having a high incidence, and high mortality, is only found in Asian countries [4]. Lineages 3, 4, 6, and 7 have been identified in only a small number of areas: lineage 3 has been mainly identified in southern China (including the Taiwan region and Hong Kong) [3,4,11,12,13], lineage 4 has been mainly identified in Japan [4], and lineages 6 and 7 have been identified in the United States [3]. Undoubtedly, lineage 1 has become the most prevalent and diverse lineage within the American and Asian swine industries [14,15]. Through 2021, lineage 1 (NADC30-like and NADC34-like) continued to be the most prevalent and diverse lineage within the U.S. swine industry [15,16,17,18]. In Peru, 75% of the strains detected were associated with PRRSV 1-7-4 strains (sublineage 1.5; NADC34-like) during 2015–2017 [19]. In South Korea and Japan, lineage 1 (sublineage 1.8; NADC30-like) comprised the second-largest population of PRRSVs [20,21]. According to the latest reports, lineage 1 (64%) strains have become the main circulating strain in China [22]. Unlike PRRSV-1 and other lineage strains of PRRSV-2, which circulate on only one continent, lineage 1 strains have a global pandemic trend. Furthermore, lineage 1C variants also threaten the global swine industry [23].

Due to the large genetic diversity and complex recombination of NADC30 strains, the pathogenicity of NADC30-like strains varies greatly [24,25,26]. As prototypes of NADC30-like strains, MN184 and NADC30 have moderate pathogenicity [27]. The NADC30-like strains in Korea show mild-to-moderate pathogenicity in challenged pigs [28,29,30]. Additionally, the Japanese strain Jpn5-37 induces moderate symptoms in animals [31]. Some NADC30-like strains in China show high pathogenicity (JL580, SD17-38, 14LY01-FJ, 14LY02-FJ, 15LY01-FJ, 15LY02-FJ, FJXS15, HBap4-2018, JS18-3) [25,32,33,34,35]; however, most strains show moderate pathogenicity (HNjz15, CHsx1401, SD53-1603, SC-d, TJnh1501, SCN17, HB17A, SCya18, HN201605, FJZ03, FJWQ16, ZJqz21, v2016/ZJ/09-03, FJ1402) [26,36,37,38,39,40,41,42,43,44]. Based on cumulative data, recombination may be responsible for the pathogenicity variance of NADC30-like PRRSVs in China, and the pathogenicity tends to be intermediate between those of the parental strains [45].

Prevention and control of PRRSV with vaccines has a long history. As early as 1994, a PRRSV-2 modified-live virus (MLV) vaccine was first commercialized in North America [46]. In China, there are currently two types of PRRS vaccines: MLV and killed virus (KV) vaccines [47]. Nine commercial vaccines are currently used to control and prevent PRRSV infection in China, including Ingelvac PRRS MLV/RespPRRS MLV, CH-1R, HuN4-F112, JXA1-P80, R98, TJM-F92, GDr180, PC, and CH-1a (KV) [48]. Of these, RespPRRS MLV and R98 are of lineage 5, and the others belong to lineage 8 [47]. KV vaccines have poor protection against homologous and heterologous strains [49], and MLV vaccines can provide adequate protection against genetically homologous strains [50]. Unfortunately, existing MLV vaccines offer only limited protection against NADC30-like strains, which are the main circulating strains in the country [37,44,47,51,52,53]. This limitation may be responsible for the rapid spread of NADC30-like PRRSVs in China; therefore, it is necessary to develop a new vaccine against NADC30-like PRRSVs. In addition, the new vaccine must be evaluated for its cross-protection effect because of the highly variable genome sequences among NADC30-like PRRSVs caused by recombination and rapid mutation. In the present study, we developed an attenuated lineage 1 PRRSV vaccine, SD-R (125th passage of strain SD in Marc-145 cells), and evaluated its homologous and heterologous protection effects. SD-R provides safe and effective protection against the homologous NADC30-like PRRSV SD and heterologous NADC30-like PRRSV HLJWK108-1711 challenge, and therefore, it can serve as an adequate vaccine against PRRSV infection in herds. To the best of our knowledge, the lineage 1 PRRSV vaccine SD-R is the first developed and evaluated attenuated NADC30-like PRRSV candidate vaccine strain in the world.

## 2. Materials and Methods

### 2.1. Ethics Statements

This study was approved by the Animal Ethics Committee of the School of Harbin Veterinary Research Institute of the Chinese Academy of Agricultural Sciences and was performed in accordance with animal ethics guidelines and approved protocols. The Animal Ethics Committee Approval Number was SYXK (Hei) 2011022.

### 2.2. Cells and Viruses

The Marc-145 cell line (an African green monkey kidney cell line) was employed for viral propagation and titration [54]. The NADC30-like PRRSV strains SD (GenBank accession number: ON254651) and HLJWK108-1711 (GenBank accession number: MN046230) were isolated and maintained in our laboratory.

### 2.3. Phylogenetic and Genomic Recombination Analysis

Multiple sequence alignments were generated using ClustalW in Lasergene (Version 7.1, DNASTAR Inc., Madison, WI, USA). Phylogenetic trees based on the whole genome were constructed in MEGA 6.0 using the neighbor-joining method with 1000 bootstrap replicates. Recombination analysis used RDP4 software with seven different algorithms (RDP, GeneConv, BootScan, MaxChi, Chimera, SiScan, and 3Seq) and SimPlot (version 3.5.1) by advancing a 500-bp sliding window along the genome alignments with a 20-bp step size.

### 2.4. Viral Culturing and Attenuation

Statistical analysis reveals that recombination hotspots range from nucleotide positions of approximately 7900 to 8100 and 12,400 to 13,500; therefore, we continuously select a lineage 1 PRRSV strain SD (with the above two recombination regions: 7365–7661 in the NSP9 region and 12305–12773 in the GP2-GP3 region) to passage in Marc-145 cells [54] using Dulbecco’s modified Eagle’s medium (DMEM), supplemented with 2% fetal bovine serum, and incubated at 37 °C with 5% CO_2_. The virus was harvested once the virus-infected Marc-145 cells showed an ~80% cytopathic effect (CPE). The titers of the wild-type strains, SD and HLJWK108-1711, and NADC30-like PRRSV SD, at different passages, were measured by seeding Marc-145 cells into 96-well cell culture plates, 2 days (d) before infection. The 50% tissue culture infective dose (TCID_50_) was calculated according to the Reed–Muench method. The 125th passage of the PRRSV strain SD was harvested and the designated lineage 1 PRRSV strain SD-R (GenBank accession number: ON254650) was characterized and evaluated in the present study.

### 2.5. Whole-Genome Sequencing of Lineage 1 PRRSV SD at Different Passages

RNA was extracted from different SD passages: F5, F6, F8, F10, F20, F30, F40, F60, F80, F100, F105, F110, F125 (SD-R), F135, and F150. Reverse transcription PCR (RT–PCR), whole-genome sequencing, genome assembly, and sequence alignments were performed as previously described [26]. Detailed information on the whole-genome amplification primers is shown in a previous article [26].

### 2.6. Safety Evaluation of High-Dose and Repeated-Dose of SD-R

Thirty 28-day-old PRRSV-free piglets (antigens of PRRSV, ASFV, CSFV, and PRV were detected using PCR or RT-PCR; antibodies of PRRSV, ASFV, CSFV, and PRV were detected using commercial ELISA kits) were obtained from a PRRS-free farm in Harbin. Fifteen PRRSV-free piglets that used to assess the safety of high-dose immunization of three batches of laboratory products were randomly divided into three groups (3B1–3B3) (Table 1). Another fifteen PRRSV-free piglets that used to assess the safety of repeated-dose immunization of three batches of laboratory products were randomly divided into three groups (3B1–3B3) and separately immunized at 0 days post-vaccination (dpv) and 14 dpv (Table 1). Each group of piglets was maintained in individual biosafety room. Clinical signs and rectal temperatures were recorded daily. Blood samples were periodically collected from individual piglets and tested for viremia. All of the piglets of the high-dose group of 3B1–3B3 were euthanized at 14 dpv. Ten tissue samples were obtained from the hearts, livers, spleens, lungs, kidneys, lymph nodes, tonsils, small intestines, bladders, and stomachs for viral detection by TaqMan^®^-based real-time fluorescence quantitative RT–PCR [55].

### 2.7. Evaluation of Immunoprotection of SD-R against Homologous and Heterologous Strains

Twenty-six 28-day-old PRRSV-free piglets (antigens of PRRSV, ASFV, CSFV, and PRV were detected using PCR or RT-PCR; antibodies of PRRSV, ASFV, CSFV, and PRV were detected using commercial ELISA kits) were obtained from two PRRS-free farms in Harbin. Thirteen PRRSV-free piglets used to assess homologous protection were randomly (piglets mixed together before the group) divided into three groups (A1–C1) (Table 2). Other PRRSV-free piglets used to test heterologous protection were randomly (piglets mixed together before the group) divided into three groups (A2–C2) (Table 2). Five piglets for each group were used for immunization and inoculation (A1, B1, A2 and B2), except for three piglets in negative control groups C1 and C2. Piglets in groups A1 and A2 were inoculated intramuscularly with 10^5.0^TCID_50_ SD-R. After 28 dpv, piglets in groups A1, B1 and A2, B2 were infected with 5th-passage SD (4 × 10^5.0^TCID_50_ per pig) and 5th-passage HLJWK108-1711 (4 × 10^5.0^TCID_50_ per pig) intramuscularly (2 mL) and intranasally (2 mL), respectively. The animals were maintained in individual biosafety rooms. Clinical signs and rectal temperatures were recorded daily. The body weights of the piglets were measured weekly. Blood samples were periodically collected from individual piglets and tested for viremia. All of the piglets were euthanized at 21 days post-inoculation (dpi). Ten tissue samples were obtained from the hearts, livers, spleens, lungs, kidneys, lymph nodes, tonsils, small intestines, bladders, and stomachs for viral detection by TaqMan^®^-based real-time fluorescence quantitative RT–PCR [55].

### 2.8. Serological Examination

Serum samples were collected at 0, 7, 14, 21, and 28 dpv and 3, 5, 7, 10, 14, and 21 dpi. PRRSV-specific antibodies were measured using a commercial ELISA kit (IDEXX, Inc., Westbrook, ME, USA) according to the manufacturer’s instructions. The PRRSV-specific antibody titer was reported as the S/P ratio, and the serum samples were considered positive if the S/P ratio was ≥ 0.4.

### 2.9. Viremia and Viral Loads in Tissue Assessment

To determine the duration of viremia and viral loads in different tissues after treatment with the SD-R vaccine strain, serum samples collected at dpv 0, 7, 14, 21, and 28 and dpi 3, 5, 7, 10, 14, and 21, and ten tissues of all the piglets were used to detect the RNA copy number of PRRSV by TaqMan^®^-based real-time fluorescence quantitative RT–PCR [55].

### 2.10. Histological Examination

At necropsy, the lungs and lymph nodes were harvested and examined for histopathology following haematoxylin and eosin (H and E) staining as previously described [26].

### 2.11. Statistical Analysis

Significant differences between two groups were determined using a *t* test (and nonparametric tests) in GraphPad 5.0 (San Diego, CA, USA). The level of significance was set at *p* < 0.05.

## 3. Results

### 3.1. Genomic Characteristics of NADC30-like PRRSV SD and HLJWK108-1711

**Figure 1:**
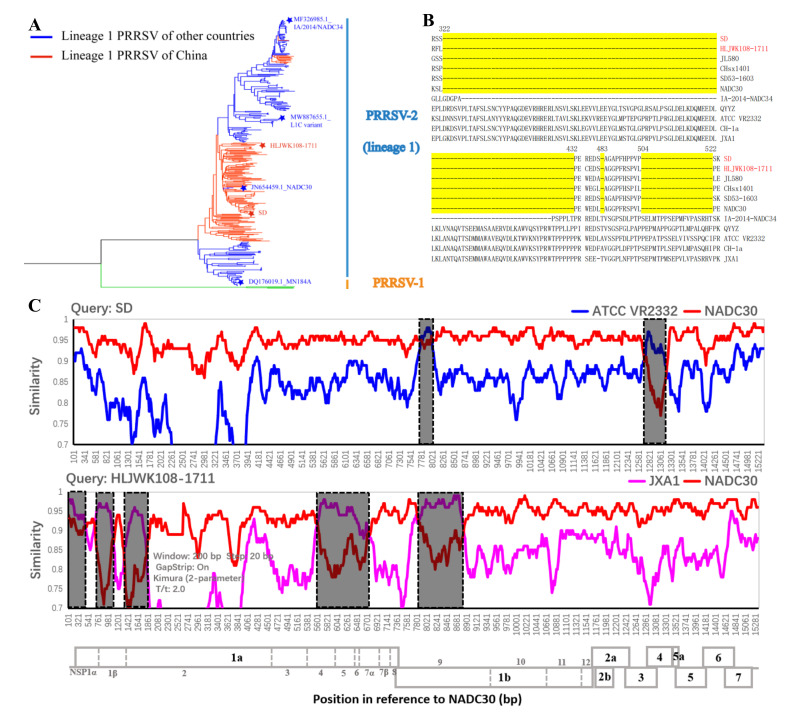
Phylogenetic analyses, NSP2-deduced amino acid alignment and recombination analyses of NADC30-like PRRSV SD and HLJWK108-1711. (**A**) Phylogenetic tree of strains SD and HLJWK108-1711 based on 341 whole genomes of lineage 1. Blue lines represent lineage 1 strains in other countries. Red lines represent lineage 1 strains in China. Both MN184A, NADC30, the L1C variant and IA/2014/NADC34 are labelled with ★. Both SD and HLJWK108-1711 are labelled with ★. (**B**) The deletion characteristics of the NSP2 protein, and 131-aa discontinuous deletions are labelled with a yellow background. (**C**) Recombination analysis of SD and HLJWK108-1711. Recombination breakpoints are shown as black dotted lines. The background color of the major parental region (NADC30) is white, whereas that of the minor parental regions (ATCC VR2332 and JXA1) is gray. NADC30, ATCC VR2332, and JXA1 are shown in red, blue, and pink, respectively.

SD and HLJWK108-1711 were isolated from two diseased pig farms in the Shandong (2016) and Heilongjiang province (2017), respectively. Phylogenetic analysis showed that the Chinese NADC30-like PRRSV formed a relatively independent branch and was closely related to NADC30 and XW018 (both strains isolated in the United States) based on a total of 344 whole genomes of lineage 1 (Figure 1A). Both SD and HLJWK108-1711 were classified into branches of NADC30-like PRRSV (L1.8/L1C) (Figure 1A). The Nsp2 proteins of SD and HLJWK108-1711 had a discontinuous 131-amino acid (aa) deletion (111 aa at position 323–433, 1 aa at position 483, and 19 aa at position 504–522) (Figure 1B). Recombination analysis revealed that both NADC30-like PRRSVs were recombinant viruses (SD: parental virus NADC30 and minor virus ATCC VR2332-like PRRSV; HLJWK108-1711: parental virus NADC30 and minor virus JXA1-like PRRSV) (Figure 1C). The recombination breakpoints of SD were observed at positions 7365, 7661, 12305, and 12,773 (ATCC-VR2332 positions 7762, 8058, 12702, and 13,170). The breakpoints separated the HLJWK108-1711 genome into ten regions, where the positions located at 521, 631, 1065, 1310, 1810, 5183, 6367, 7488, 8443 (JXA1 positions 524, 634, 1068, 1313, 1813, 5490, 6674, 7795 and 8750) (Figure 1C); however, the genomic nucleotide similarity between SD-R and HLJWK108-1711 was 89.9%, and the nucleotide similarity of the skeleton section of NADC30 was only 91.4% (Table 3). The nucleotide and amino acid similarity among different genes between SD-R and HLJWK108-1711 were 82.7–97.7% and 80.2–100%, respectively (Table 3).

### 3.2. Nucleotide and Amino Acid Mutations of Different SD Passages

To develop a live attenuated lineage 1 PRRSV vaccine, we first isolated a SD strain and passaged it in Marc-145 cells. Compared with the parental virus SD, there were 75 nucleotide changes at the 125th passage (Table S1). Among these mutations were two nucleotide changes (at position 29 (C–T) and 36 (C–T)) in the 5′-UTR and two (at position 22 (T–C) and 69 (T–C)) in the 3′-UTR (Table S1). Other nucleotide mutations were observed in Nsps and structural proteins, 31 of which were missense mutations, causing a change in 31 amino acids in Nsp2-5, 9-12, GP2-5, M, N, and ORF5a (Table S1). The major changes in amino acids were located on Nsp2 and minor structural proteins GP2-4 (Table S1). No nucleotide amino acid changes were observed from passages 125 through 150 (Table S1).

### 3.3. Both High-Dose and Repeated-Dose Tests of SD-R Are Safe for Piglets

**Figure 2:**
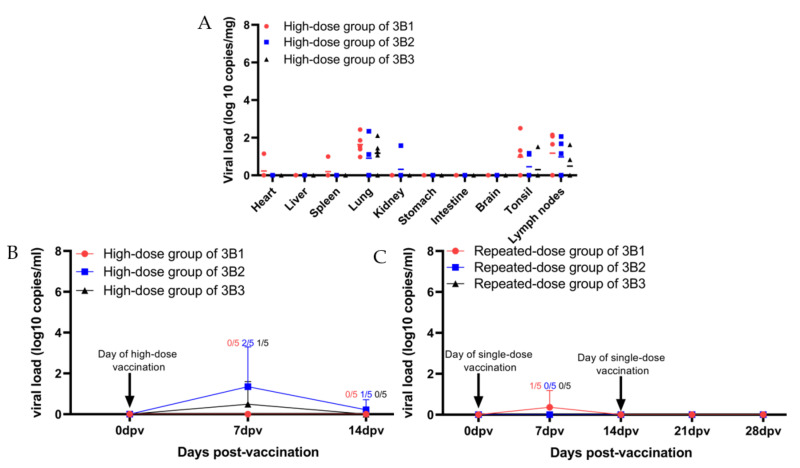
Viral loads in ten tissues or sera of high-dose groups (**A**) and (**B**) and repeated-dose (**C**) groups. PRRSV viral RNA in sera and tissues was measured by qPCR. High-dose groups and repeated-dose groups of three batches of laboratory products of SD-R were labeled with a red circle (●), blue box (■), and black triangle (▲).

Over the course of the study, the piglets in high-dose group of 3B1–3B3 and repeated-dose group of 3B1–3B3 had no clinical signs of PRRSV and no fever (data not shown). To evaluate viremia and the distribution of PRRSV in different tissues, the serum samples and ten organ tissues were evaluated by real-time PCR, which were collected either from 0, 7, 14 dpv in high-dose groups or from 0, 7, 14, 21 and 28 dpv in repeated-dose groups. The low-level viral copy of the serum samples at 7 or 14 dpv (2 out of 5 piglets at 7 dpv and 1 out of 5 piglets at 14 dpv of the high-dose group of 3B2; 1 out of 5 piglets at 7 dpv of high-dose group of 3B3; 1 out of 5 piglets at 7 dpv of the repeated-dose group of 3B1) were detected (Figure 2B,C). In addition, the low-level viral copy was detected in lungs, tonsils, lymph nodes, and a small amount of tissues (heart and spleen in the high-dose group of 3B1; kidney in high-dose group of 3B3) (Figure 2A).

### 3.4. Clinical Reactions after Immunization and Challenge

**Figure 3:**
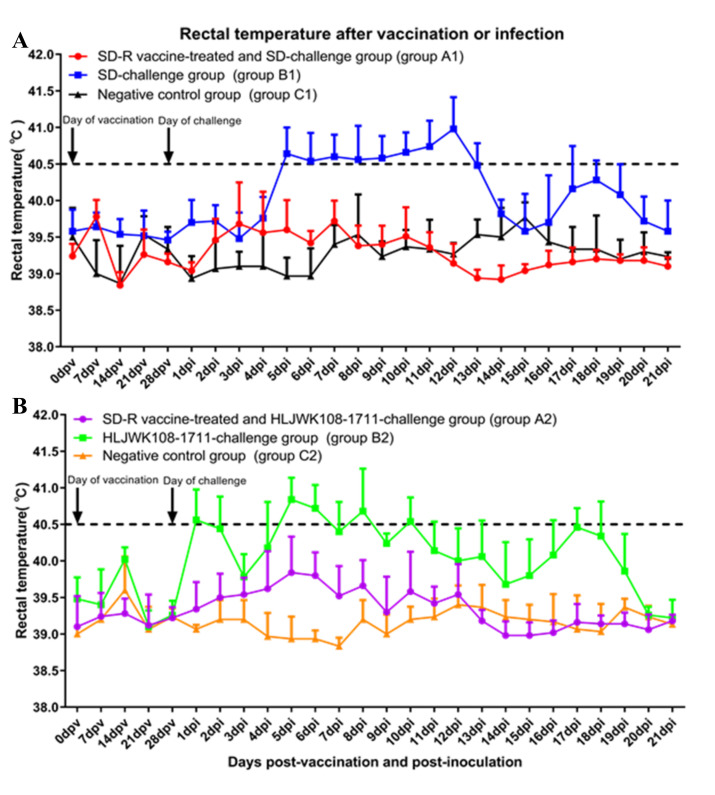
Rectal temperatures after SD-R immunization and challenge with parental virus SD (**A**) and NADC30-like PRRSV HLJWK108-1711 (**B**). Rectal temperatures ≥ 40.5 °C were defined as fever. The group A1, B1 and C1 are labelled with red, blue, and black, respectively. The group A2, B2, and C2 are labelled with purple, green, and orange, respectively. The arrows mark the time of the immunization and challenge.

**Figure 4:**
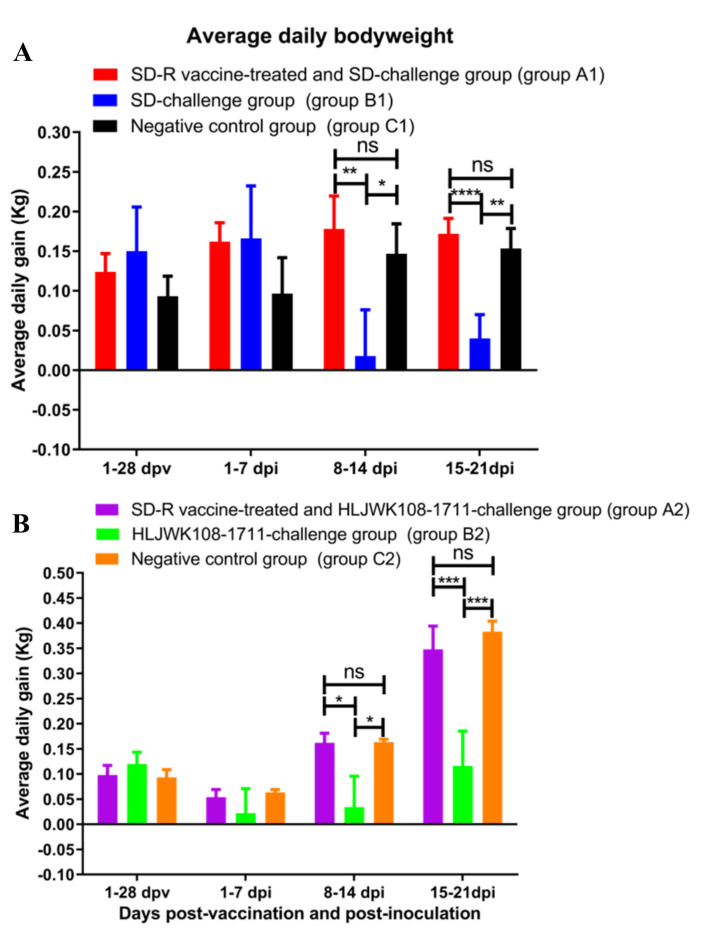
Effects after SD−R immunization and challenge with parental virus SD (**A**) and NADC30−like PRRSV HLJWK108−1711 (**B**) on the weight of the piglets. All the experimental piglets were weighed at 0 and 28 dpv and 7, 14, and 21 dpi. The average daily gain of 5 or 3 piglets per group was calculated for days 0 to 28 dpv and 1 to 7 dpi, 8 to 14 dpi, and 15 to 21 dpi. The bars represent the average daily body weight of five or three piglets ± SD. An asterisk (*) indicates a significant difference between the group A1 and B1, B1 and C1 or the group A2 and B2, B2 and C2 (****, *p* < 0.0001; ***, *p* < 0.001; **, *p* < 0.01; *, *p* < 0.05; ns, *p* > 0.05). Groups A1, B1, and C1 are labelled red, blue, and black, respectively. Groups A2, B2, and C2 are labelled purple, green, and orange, respectively.

After immunization, none of the piglets in groups A1 (SD-R vaccine-treated and SD-challenge group) and A2 (SD-R vaccine-treated and HLJWK108-1711-challenge group) showed any clinical signs of PRRS compared with the negative control group and challenge control group. After the challenge, none of the piglets in groups A1 (Figure 3A and Figure 4A) and A2 (Figure 3B and Figure 4B) showed significant changes in body temperature or weight loss. The piglets in group B1 (nonvaccinated and SD-challenge group) had various disease manifestations at 5 dpi, including fever persisting for 6–13 d (≥ 40.5 °C) (Figure 3A), and the piglets in group B2 (nonvaccinated and HLJWK108-1711-challenge group) had various disease manifestations as early as 1 dpi, including intermittent fever for 5–10 d (≥ 40.5 °C) (Figure 3B). In addition, the piglets in groups B1 and B2 had various levels of anorexia and emaciation (data not shown). Compared with the negative control piglets in groups C1 and C2, the piglets in groups B1 and B2 gained less body weight (*p* < 0.05) during 8–14 dpi and 15–21 dpi (Figure 4A,B). Over the course of the study, the piglets in groups C1 and C2 had no clinical signs of disease.

### 3.5. Antibody Responses in Immunized or Challenged Piglets

**Figure 5:**
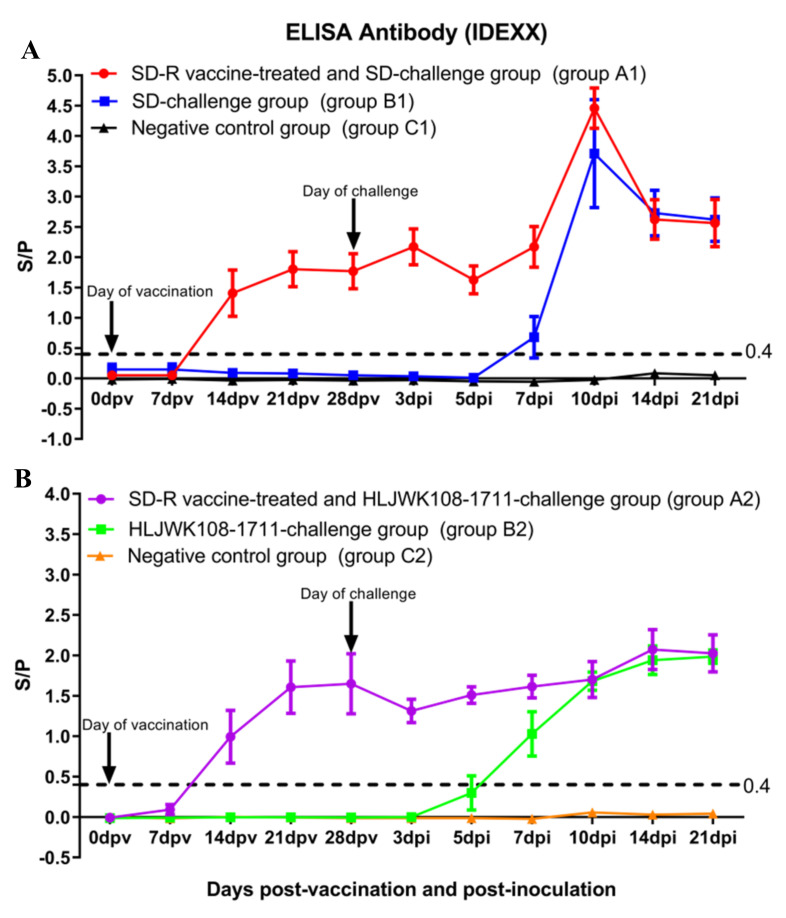
Anti−PRRSV antibody levels after SD−R immunization and challenge with parental virus SD (**A**) and NADC30−like PRRSV HLJWK108−1711 (**B**). Solid line, threshold value above ≥ 0.4, in which titers were considered positive for anti−PRRSV antibodies. The groups A1, B1, and C1 are labelled with red, blue, and black, respectively. The groups A2, B2, and C2 are labelled with purple, green, and orange, respectively.

The antibody response in the ELISA showed that all immunized piglets in groups A1 and A2 were seroconverted by 14 dpv (Figure 5A,B). A total of 3 of 5 piglets in group B1 were seroconverted by 7 dpi, and the remaining piglets seroconverted by 10 dpi (Figure 5A). Two of five piglets in group B2 were seroconverted by 5 dpi, and the remaining piglets seroconverted by 7 dpi (Figure 5B). No PRRSV-specific antibodies were detected in the control piglets prior to challenge (Figure 5A,B). Antibody responses of piglets in groups C1 and C2 were negative throughout the study (Figure 5A,B).

### 3.6. Viremia and Viral Tissue Distribution between the Immunized-Challenge Group and the Challenge Group

**Figure 6:**
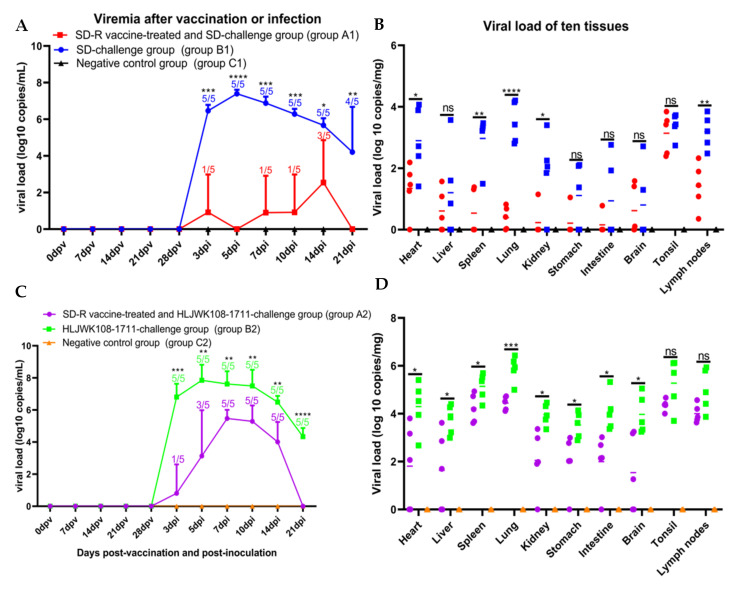
Viremia and viral loads in ten tissues after SD-R immunization and challenge with parental virus SD ((**A**) and (**B**)) and NADC30-like PRRSV HLJWK108-1711 ((**C**) and (**D**)). PRRSV viral RNA in sera and tissues were measured by qPCR. The groups A1, B1, and C1 are labelled with red, blue, and black, respectively. The groups A2, B2, and C2 are labelled with purple, green, and orange, respectively. An asterisk (*) indicates a significant difference between the group A1 and B1 or the group A2 and B2 (ns, *p* > 0.5; *, *p* < 0.05; **, *p* < 0.01; ***, *p* < 0.001; ****, *p* < 0.0001). The numbers represent the number of piglets with viral loads and the number of piglets in this group.

To further evaluate the difference in viremia and distribution in ten tissues among different groups, serum samples from 0, 7, 14, 21, and 28 dpv, 3, 5, 7, 10, 14, and 21 dpi and ten organ tissues were evaluated using real-time PCR. The RNA copy numbers of the serum samples in groups B1 and B2 reached their highest levels at 5 dpi and then gradually declined in these two groups (Figure 6A,C). The viremia levels at every time point in groups A1 and A2 were significantly lower than those in groups B1 and B2, respectively (Figure 6A,C). Furthermore, the viremia levels and the number of pigs with viremia in the homologous protection group (groups A1 and B1) were significantly lower than those in the heterologous protection group (groups A2 and B2) (Figure 6A,C). The viral loads of ten tissues in groups A1 (except liver, stomach, intestine, brain, and tonsil) and A2 (except tonsil and lymph nodes) were significantly lower than those in groups B1 and B2, respectively (Figure 6B,D).

### 3.7. Gross Pathological and Histopathological Changes

**Figure 7:**
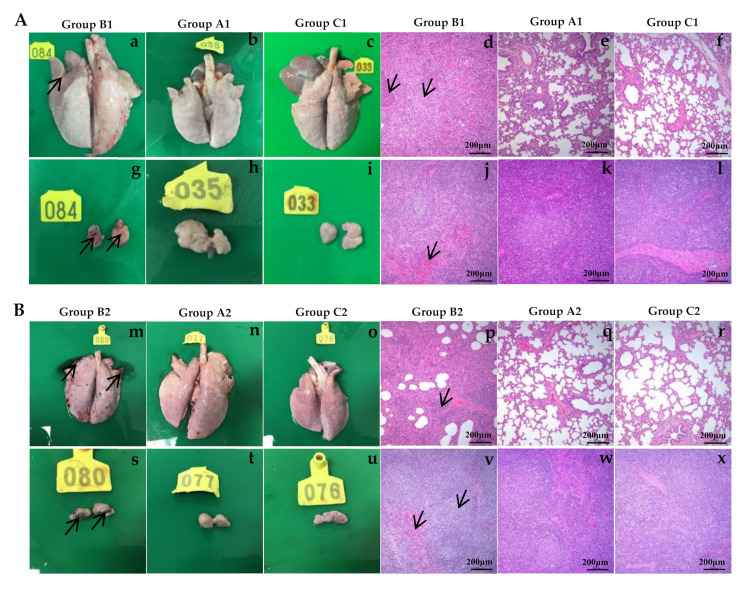
Gross and histological lesions of lungs and lymph nodes from SD-R immunization and challenge with parental virus SD group (**A**) and NADC30-like PRRSV HLJWK108-1711 group (**B**). Consolidation and/or oedema in the lungs in groups B1 and B2 (a and m), and hemorrhage in the lymph nodes in groups B1 and B2 (g and s) were observed when compared with groups A1 and A2 (b and n; h and t) and groups C1 and C2 (c and o; i and u). Compared with groups A1 and A2 (e and q) and groups C1 and C2 (f and r), a large amount of inflammatory cell infiltration, epithelial cell proliferation and significant alveolar diaphragm widening in the lungs were observed in groups B1 and B2 (d and p). Compared with groups A1 and A2 (k and w) and groups C1 and C2 (l and x), mild lymphocytopenia and medullary hemorrhage were observed in parts of the lymphoid nodules in groups B1 and B2 (j and v).

Compared with the piglets in the immunized-challenge groups (A1 and A2) (Figure 7(Ab,Ah) and Figure 7(Bn,Bt)) and negative control groups (C1 and C2) (Figure 7(Ac,Ai,Bo,Bu)), the piglets in the challenge groups (B1 and B2) showed lesions typical of PRRS, such as consolidation in the lungs and hemorrhaging in the lymph nodes (Figure 7(Aa,Ag,Bm,Bs)). Histopathology revealed a large amount of inflammatory cell infiltration, epithelial cell proliferation, significant alveolar diaphragm widening in the lungs (Figure 7(Ad,Bp), and decreased lymphocyte and medullary bleeding in the lymph nodes (Figure 7(Aj,Bv)) in the challenge groups (B1 and B2), compared with the negative control groups (Figure 7(Af,Al,Br,Bx)). Notably, there was almost no pathological damage to the lungs (Figure 7(Ae,Bq)) and only mildly decreased levels of lymphocytes in the immunized-challenge groups (A1 and A2) (Figure 7(Ak,Bw)).

## 4. Discussion

Lineage 1, especially NADC30-like PRRSV, has become the most prevalent PPRSV lineage in North America and Asia [14,15,16,17,18,19,20,21,22]. In 2013, a new PRRSV strain called NADC30-like PRRSV, which has a unique 131-aa deletion within its NSP2 protein, was isolated from diseased piglets in China [32,56]. This PRRSV originated in the United States and has become one of the major endemic strains in China since 2016 [57]. The pathogenicity of NADC30-like PRRSVs ranges from moderate [26,36,38] to high [32,33,58], and most of them are moderately pathogenic [26,36,37,38,39,40,41,44,47,48,53]; however, the current commercial vaccines, Ingelvac PRRS MLV/RespPRRS MLV [44,52], CH-1a [47], HuN4-F112 [51], JXA1-P80 [52], R98 [53], TJM-F92 [51], and GDr180 [26,51], do not provide completely effective protection against NADC30-like PRRSVs. Here, we selected the moderately pathogenic NADC30-like PRRSV SD strain and described a newly developed lineage 1 PRRSV vaccine candidate, SD-R, which is efficacious in the prevention of clinical infection caused by NADC30-like PRRSVs.

Unlike CH-1a or HP-PRRSV, NADC30-like PRRSVs have lower levels of whole-genome similarity [26,57] and a wider variety of recombination patterns [16,26]. Almost all NADC30-like PRRSVs are recombinant viruses [16,26]. Although recombination breakpoints are relatively random, statistical analysis reveals that recombination hotspots range from nucleotide positions of approximately 7900 to 8100 (Nsp9) and 12,400 to 13,500 (GP2-GP3) [16,26]. The recombination of PRRSV at these regions might be associated with an increase in replication capacity and cellular tropism, which presumably makes it easier to survive and spread, and ultimately drives the pathogenesis of PRRSV [16]; therefore, we selected an NADC30-like PRRSV SD with the above two recombination regions (7365–7661 in the NSP9 region and 12305–12773 in the GP2–GP3 region) for passage in Marc-145 cells.

In the present study, SD-R, a genetically stable attenuated viral strain in Marc-145 cells, was obtained by serial passaging in Marc-145 cells with the lineage 1 PRRSV SD strain. All amino acid mutations related to SD were observed before the 125th passage. No nucleotide and amino acid mutations were observed between the 125th and 150th passages. This indicated that the SD strain at the 125th passage had adapted to the Marc-145 cells and was subsequently stably passaged. The commercial HP-PRRSV vaccines JXA1-R, TJM, HuN4-F112, and GDr180 were obtained through passaging in Marc-145 cells for 80, 92, 112, and 180 passages, respectively [54,59,60,61]. As the pathogenicity of SD is far lower than that of HP-PRRSV, we speculate that SD-R (the 125th passage in Marc-145 cells) is safer for piglets than commercial HP-PRRSV vaccines. Furthermore, both high-dose and repeated-dose tests based on SD-R were safe for all piglets, including no fever, no clinical symptoms and a small number of immunized piglets detected with viremia and viruses in lungs, tonsils, and lymph nodes. The duration of viremia varied in different PRRS vaccines with different immune doses [54,59]. Piglets were immunized with HP-PRRS vaccines at a dose of 10^5.0^TCID_50_. The viremia duration of some pigs was about 3 to 21 days (JXA1-P80: 3~14d; HuN4-F112: 7~14d; TJM-F92: 3~21d) [54,59,60]. In piglets immunized with a classical PRRSV vaccine (CH-1R), viremia could not be detected [62,63]. We speculated that SD-R, similarly to CH-1R, has higher safety for piglets.

In this study, piglets immunized with SD-R developed a rapid and effective humoral response and were effectively protected against the NADC30-like PRRSV challenge. Indeed, PRRSV vaccines have poor cross-protection effects [45,51]. Most studies have found that MLV cannot provide complete cross-protection in NADC30-like infected piglets, including shortening the period of fever with fewer pig numbers of clinical manifestations [37,44,47,51,52,53], only improving body weight gain at some point in the study [37,44,48,52], without decreasing the viremia or viral loads in tissues [51,64], and pathological lesions in lung and lymphoid tissues [37,47,51,52]; however, surprisingly, SD-R could provide better cross-protection (including no fever, a significant improvement in weight gain, a significantly reduced viral load in blood and tissues, and no visible pathological damage) (Table S2), even though the genomic nucleotide similarity of SD and HLJWK108-1711 was only 89.9%. All the piglets immunized with SD-R and then challenged with SD or HLJWK108-1711 survived without any major clinical signs at any point in the experimental period. Piglets in the immunized and challenge groups were healthier than those in the challenge control group based on clinical signs, body temperature, body weight, viremia, and viral loads in tissues. Altogether, these results suggested that the SD-R candidate vaccine is effective against infections caused by different NADC30-like PRRSVs; however, the detailed molecular basis of cross-protection induced by the SD-R vaccine and its attenuation mechanism remain unclear. Furthermore, the cross-protection against other types of PRRSVs, such as NADC34-like PRRSV, QYYZ-like PRRSV, and HP-PRRSV, should be studied further.

## 5. Conclusions

In conclusion, we developed the first attenuated lineage 1 PRRSV candidate vaccine strain, SD-R. Furthermore, SD-R was sufficiently attenuated and antigenic enough to confer clinical protection against the homologous and heterologous NADC30-like PRRSV challenges.

## Figures and Tables

**Table 1:** Group information for safety evaluation of SD-R.

Groups	Vaccination	Number of Animals
High-dose group of 3B1	2 × 10^6.0^TCID_50_	5 (100; 101;102; 103; 104)
High-dose group of 3B2	2 × 10^6.5^TCID_50_	5 (105; 106; 107; 108; 109)
High-dose group of 3B3	2 × 10^6.2^TCID_50_	5 (110; 111; 112; 113; 114)
Repeated-dose group of 3B1	2 × 10^5.0^TCID_50_ +2 × 10^5.0^TCID_50_	5 (70; 71; 73; 74; 75)
Repeated-dose group of 3B2	2 × 10^5.5^TCID_50_ +2 × 10^5.5^TCID_50_	5 (77; 78; 79; 80; 81)
Repeated-dose group of 3B3	2 × 10^5.2^TCID_50_ +2 × 10^5.2^TCID_50_	5 (92; 93; 94; 95; 96)

**Table 2:** Group information for effectiveness evaluation of SD-R.

Groups	Corresponding Groups	Number of Animals	Vaccination	Challenge
Group A1	SD-R vaccine-treated and SD-challenge group	5 (031; 032;035; 037; 038)	2 × 10^5.0^TCID_50_ per pig (SD-R)	4 × 10^5.0^TCID_50_ per pig (SD)
Group B1	SD-challenge group	5 (081; 082; 083; 084; 085)	DMEM	
Group C1	Negative control group	3 (033; 034; 036)		DMEM
Group A2	SD-R vaccine-treated and HLJWK108-1711-challenge group	5 (073; 074; 075; 077; 079)	2 × 10^5.0^TCID_50_ per pig (SD-R)	4 × 10^5.0^TCID_50_ per pig (HLJWK108-1711)
Group B2	HLJWK108-1711-challenge group	5 (057; 058; 059; 078; 080)	DMEM	
Group C2	Negative control group	3 (076; 088; 090)		DMEM

**Table 3:** The nucleotide and deduced amino acid similarity between SD and HLJWK108-1711.

Gene	Nucleotide Similarity (%)	Deduced Amino Acid Similarity (%)	Gene	Nucleotide Similarity (%)	Deduced Amino Acid Similarity (%)
Whole genome	89.9	/	Nsp8	94.8	93.3
skeleton section of NADC30	91.4	/	Nsp9	89.2	97.2
5′UTR	91.5	/	Nsp10	94.0	97.3
3′UTR	97.3	/	Nsp11	93.1	95.1
Nsp1α	88.5	93.3	Nsp12	93.7	96.1
Nsp1β	82.7	80.2	ORF2a	94.0	92.6
Nsp2	87.0	84.9	ORF2b	97.7	100
Nsp3	90.4	93.0	ORF3	86.8	84.3
Nsp4	82.8	92.6	ORF4	94.6	96.1
Nsp5	84.1	91.8	ORF5	93.5	93.0
Nsp6	93.8	100	ORF5a	96.4	95.7
Nsp7α	91.1	90.6	ORF6	95.6	98.9
Nsp7β	90.9	90.9	ORF7	94.9	93.5

## Data Availability

The datasets generated or analyzed during this study are available from the corresponding author on reasonable request.

## Supplementary Materials

The following are available online at https://www.mdpi.com/article/10.3390/vaccines10050752/s1, Table S1: Nucleotide and amino acid changes in 16 different mutant passages; Table S2: Comparison of protective effect of different vaccine strains or candidate vaccine strain against NADC30-like PRRSVs.
