# Supplementary material for: Lineage 1 Porcine Reproductive and Respiratory Syndrome Virus Attenuated Live Vaccine Provides Broad Cross-Protection against Homologous and Heterologous NADC30-Like Virus Challenge in Piglets
Source: Vaccines (Basel). 2022 May 10;10(5):752. doi: 10.3390/vaccines10050752 (PMC9146329; doi:10.3390/vaccines10050752)
Supplement: Supplementary file 1 [file vaccines-10-00752-s001.zip › vaccines-1679929-supplementary.pdf]

Supplementary Materials

Table S1 Nucleotide and amino acid changes in 16 different mutant passages.

NSP	Position	SD	Mutation at indication no. of passages															
			5th	6th	8th	10th	20th	30th	40th	60th	80th	100th	105th	110th	125th	135th	150th
5'UTR	29	C	C	C	C	C	C	C	C	C	T	T	T	T	T	T	T
	36	C	C	C	C	C	C	C	C	T	T	T	T	T	T	T	T
NSP1α	504	C	C	C	C	C	C	C	C	C	C	C	T	T	T	T	T
NSP1β	229	T	T	T	T	T	T	T	T	T	C	C	C	C	C	C	C
NSP2	21	A	A	A	A	A	A	A	A	A	A	A	A	A	G	G	G
	63	C	C	C	C	C	C	C	C	T	T	T	T	T	T	T	T
	565	G	G	G	G	G	G	G	A	A	A	A	A	A	A	A	A
	567	G	G	G	G	G	G	G	G	T	T	T	T	T	T	T	T
	621	T	T	T	C	C	C	C	C	C	C	C	C	C	C	C	C
	901	C	C	C	C	C	C	C	A	A	A	A	A	A	A	A	A
	1134	C	C	C	C	C	C	C	T	T	T	T	T	T	T	T	T
	1357	G	G	G	G	G	A	A	A	A	A	A	A	A	A	A	A
	1546	G	G	G	G	G	G	G	C	C	C	C	C	C	C	C	C
	2000	C	C	C	C	C	C	C	C	T	T	T	T	T	T	T	T
	2184	T	T	T	T	T	T	T	T	T	C	C	C	C	C	C	C
	2257	T	T	T	T	T	T	T	T	T	C	C	C	C	C	C	C
	2439	A	A	A	A	A	A	A	A	A	G	G	G	G	G	G	G
	2634	A	A	A	A	G	G	G	G	G	G	G	G	G	G	G	G
	2817	C	C	C	C	C	C	C	C	A	A	A	A	A	A	A	A
	3117	C	C	C	C	C	C	C	C	T	T	T	T	T	T	T	T
NSP3	254	T	T	T	T	T	T	T	C	C	C	C	C	C	C	C	C
NSP4	257	G	G	G	G	A	A	A	A	A	A	A	A	A	A	A	A
	309	C	C	C	C	T	T	T	T	T	T	T	T	T	T	T	T
NSP5	261	A	A	A	A	T	T	T	T	T	T	T	T	T	T	T	T
	293	T	T	T	T	T	T	T	T	C	C	C	C	C	C	C	C
	492	C	C	T	T	T	T	T	T	T	T	T	T	T	T	T	T
NSP7α	192	C	C	C	C	C	C	T	T	T	T	T	T	T	T	T	T
	240	C	C	T	T	T	T	T	T	T	T	T	T	T	T	T	T
NSP9	69	G	G	G	G	G	A	A	A	A	A	A	A	A	A	A	A
	361	G	G	G	G	A	A	A	A	A	A	A	A	A	A	A	A
	468	G	G	G	G	A	A	A	A	A	A	A	A	A	A	A	A
	1194	T	T	T	T	C	C	C	C	C	C	C	C	C	C	C	C
	1312	T	T	T	T	C	C	C	C	C	C	C	C	C	C	C	C
	1419	A	A	A	A	G	G	G	G	G	G	G	G	G	G	G	G
	1626	A	A	A	A	A	A	A	A	T	T	T	T	T	T	T	T
NSP10	87	C	C	C	C	T	T	T	T	T	T	T	T	T	T	T	T
	294	C	C	C	C	T	T	T	T	T	T	T	T	T	T	T	T
	577	G	G	G	G	A	A	A	A	A	A	A	A	A	A	A	A
	867	A	A	A	A	A	A	A	A	G	G	G	G	G	G	G	G
NSP11	234	T	T	T	T	T	T	T	T	T	T	C	C	C	C	C	C
	651	A	A	A	A	G	G	G	G	G	G	G	G	G	G	G	G
NSP12	76	T	T	T	T	T	T	T	T	C	C	C	C	C	C	C	C
	174	C	C	C	C	C	C	C	C	T	T	T	T	T	T	T	T
	373	A	A	A	A	A	G	G	G	G	G	G	G	G	G	G	G
ORF2a	146	A	A	A	A	A	A	A	A	A	A	C	C	C	C	C	C
	289	A	A	A	G	G	G	G	G	G	G	G	G	G	G	G	G
	292	T	T	T	C	C	C	C	C	C	C	C	C	C	C	C	C
	345	G	G	G	G	G	G	G	G	A	A	A	A	A	A	A	A
	507	A	A	A	A	A	A	A	A	A	G	G	G	G	G	G	G
	705	T	T	T	T	T	T	T	T	T	C	C	C	C	C	C	C
	740	C	C	C	C	C	T	T	T	T	T	T	T	T	T	T	T
ORF2b	141	A	A	A	A	A	A	A	A	A	A	C	C	C	C	C	C
ORF3	82	T	T	T	T	T	T	T	T	T	C	C	C	C	C	C	C
	117	C	C	C	C	C	T	T	T	T	T	T	T	T	T	T	T
	164	C	C	C	C	C	C	C	C	T	T	T	T	T	T	T	T
	272	G	G	G	G	G	G	G	G	G	G	A	A	A	A	A	A
	286	C	C	C	C	C	C	C	C	C	T	T	T	T	T	T	T
ORF4	340	T	T	T	A	A	A	A	A	A	A	A	A	A	A	A	A
	370	A	A	A	A	A	G	G	G	G	G	G	G	G	G	G	G
	479	A	A	A	A	A	A	A	C	C	C	C	C	C	C	C	C
ORF5a	7	A	A	A	A	A	A	A	G	G	G	G	G	G	G	G	G
ORF5	174	C	C	C	C	A	A	A	A	A	A	A	A	A	A	A	A
	271	G	G	G	G	G	G	G	G	G	A	A	A	A	A	A	A
	330	T	T	T	T	T	T	T	T	C	C	C	C	C	C	C	C
ORF6	55	T	T	T	T	C	C	C	C	C	C	C	C	C	C	C	C
	217	C	C	C	C	C	C	C	C	C	T	T	T	T	T	T	T
	396	A	A	A	A	A	A	A	A	G	G	G	G	G	G	G	G
ORF7	21	G	G	G	G	A	A	A	A	A	A	A	A	A	A	A	A
	76	T	T	T	T	C	C	C	C	C	C	C	C	C	C	C	C
	82	A	A	A	A	A	A	A	G	G	G	G	G	G	G	G	G
	171	A	A	A	A	A	A	A	A	A	A	G	G	G	G	G	G
	177	T	T	T	T	T	T	T	T	C	C	C	C	C	C	C	C
	363	C	C	C	C	C	C	C	C	C	C	T	T	T	T	T	T
3'UTR	22	T	T	T	T	T	T	T	T	T	T	C	C	C	C	C	C
	69	T	T	T	T	C	C	C	C	C	C	C	C	C	C	C	C
NSP1β	77	Y	Y	Y	Y	Y	Y	Y	Y	Y	H	H	H	H	H	H	H
NSP2	301	Q	Q	Q	Q	Q	Q	Q	K	K	K	K	K	K	K	K	K

	453	A	A	A	A	A	**T**	**T**	**T**	**T**	**T**	**T**	**T**	**T**	**T**	**T**	**T**
	516	G	G	G	G	G	G	G	**R**	**R**	**R**	**R**	**R**	**R**	**R**	**R**	**R**
	667	A	A	A	A	A	A	A	A	**V**	**V**	**V**	**V**	**V**	**V**	**V**	**V**
	1020	C	C	C	C	C	C	C	C	C	**S**	**S**	**S**	**S**	**S**	**S**	**S**
NSP3	85	V	V	V	V	V	V	V	**A**	**A**	**A**	**A**	**A**	**A**	**A**	**A**	**A**
NSP4	86	G	G	G	G	**E**	**E**	**E**	**E**	**E**	**E**	**E**	**E**	**E**	**E**	**E**	**E**
NSP5	98	V	V	V	V	V	V	V	V	**A**	**A**	**A**	**A**	**A**	**A**	**A**	**A**
NSP9	121	V	V	V	V	**I**	**I**	**I**	**I**	**I**	**I**	**I**	**I**	**I**	**I**	**I**	**I**
NSP10	193	A	A	A	A	**T**	**T**	**T**	**T**	**T**	**T**	**T**	**T**	**T**	**T**	**T**	**T**
NSP11	60	Y	Y	Y	Y	Y	Y	Y	Y	Y	Y	**C**	**C**	**C**	**C**	**C**	**C**
	188	T	T	T	T	T	T	T	T	T	T	**A**	**T**	**A**	**A**	**A**	**A**
NSP12	125	K	K	K	K	K	**E**	**E**	**E**	**E**	**E**	**E**	**E**	**E**	**E**	**E**	**E**
	49	Y	Y	Y	Y	Y	Y	Y	Y	Y	Y	**S**	**S**	**S**	**S**	**S**	**S**
	97	M	M	M	M	**V**	**V**	**V**	**V**	**V**	**V**	**V**	**V**	**V**	**V**	**V**	**V**
GP2a	98	F	F	F	F	**L**	**L**	**L**	**L**	**L**	**L**	**L**	**L**	**L**	**L**	**L**	**L**
	115	M	M	M	M	M	M	M	M	**I**	**I**	**I**	**I**	**I**	**I**	**I**	**I**
	247	S	S	S	S	S	**L**	**L**	**L**	**L**	**L**	**L**	**L**	**L**	**L**	**L**	**L**
	28	S	S	S	S	S	S	S	S	S	**P**	**P**	**P**	**P**	**P**	**P**	**P**
GP3	55	P	P	P	P	P	P	P	P	**L**	**L**	**L**	**L**	**L**	**L**	**L**	**L**
	91	G	G	G	G	G	G	G	G	G	G	**E**	**E**	**E**	**E**	**E**	**E**
	96	P	P	P	P	P	P	P	P	P	**S**	**S**	**S**	**S**	**S**	**S**	**S**
	114	F	F	F	F	**I**	**I**	**I**	**I**	**I**	**I**	**I**	**I**	**I**	**I**	**I**	**I**
GP4	124	I	I	I	I	I	**V**	**V**	**V**	**V**	**V**	**V**	**V**	**V**	**V**	**V**	**V**
	160	E	E	E	E	E	E	E	**A**	**A**	**A**	**A**	**A**	**A**	**A**	**A**	**A**
ORF5a	3	R	R	R	R	R	R	R	**G**	**G**	**G**	**G**	**G**	**G**	**G**	**G**	**G**
GP5	58	N	N	N	N	**K**	**K**	**K**	**K**	**K**	**K**	**K**	**K**	**K**	**K**	**K**	**K**
	91	G	G	G	G	G	G	G	G	G	**S**	**S**	**S**	**S**	**S**	**S**	**S**
M	73	L	L	L	L	L	L	L	L	L	**F**	**F**	**F**	**F**	**F**	**F**	**F**
N	28	K	K	K	K	K	K	K	**E**	**E**	**E**	**E**	**E**	**E**	**E**	**E**	**E**

Table S2 Comparison of protective effect of different vaccine strains or candidate vaccine strain against NADC30-like PRRSVs.

Vaccine strains or candidate vaccine strain	The days post-vaccination (before virus challenge) (dpv)	NADC30-like PRRSV strain	The days post-challenge (dpc)	Evaluation parameters and grouping			Refference
				Parameters of evaluation	Challenge group	Immunized group	
Ingelvac PRRS® MLV	28	TJnh1501	21	Clinical symptoms	Obvious clinical symptoms	Could not alleviate the clinical signs	[52]
				Days of fever	12 (>40.0℃)	3 (>40.0℃)	
				Average daily gain	Significantly lower than negative control group	Significantly lower than negative control group	
				Pathological and histopathological lesions	Moderate histopathological lesions	Could not reduce lung gross and microscopic lesions	
				Viremia	High viremia	Significantly reduced at some point in time.	
JXA1-R	28	TJnh1501	21	Clinical symptoms	Obvious clinical symptoms	Could not alleviate the clinical signs	
				Days of fever	12 (>40.0℃)	9 (>40.0℃)	
				Average daily gain	Significantly lower than negative control group	Significantly lower than negative control group	
				Pathological and histopathological lesions	Moderate histopathological lesions	Could not reduce lung gross and microscopic lesions	
				Viremia	High viremia	Significantly reduced at some point in time.	
Commercial vaccine 1 and 3	28	HNjz15	14	Clinical symptoms	PRRSV-specific clinical symptoms (5/5)	Could not alleviate the clinical signs	[51]
				Days of fever	4 (≧40.0℃)	1~2 (≧40.0℃)	
				Average daily gain	Significantly lower than negative control group	Significantly lower than negative control group	
				Pathological and histopathological lesions	Typical pathological lesions	The lesions were reduced but not significant.	
				Viremia	High viremia	Significantly reduced	
Commercial vaccine 2, 4 and 5	28	HNjz15	14	Clinical symptoms	PRRSV-specific clinical symptoms (5/5)	PRRSV-specific clinical symptoms (2~3/5)	
				Days of fever	4 (≧40.0℃)	1~2 (≧40.0℃)	
				Average daily gain	Significantly lower than negative control group	Significantly lower than negative control group	
				Pathological and histopathological lesions	Typical pathological lesions	The lesions were reduced but not significant.	
				Viremia	High viremia	No significant reduction	
TJM-F92	28	HN201605	21	Clinical symptoms	Mild clinical symptoms	No clinical symptoms	[43]
				Days of fever	7 (>40℃)	No fever	
				Average daily gain	Significantly lower than negative control group	No significant difference compared with negative control group	
				Pathological and histopathological lesions	Fewer pathological changes	No visible gross pathological changes	

				Viremia	High viremia	No viremia	
TJM-F92	28	FJ1402	14	Clinical symptoms	Severe clinical symptoms	/	[53]
				Days of fever	10 (⩾40.0℃)	No fever	
				Average daily gain	/	/	
				Pathological and histopathological lesions	Severe pathological damage	Mild pathological damage	
				Viremia	High viremia	Significantly reduced at some point in time.	
R98	28	FJ1402	14	Clinical symptoms	Severe clinical symptoms	/	
				Days of fever	10 (⩾40.0℃)	7 (⩾40.0℃)	
				Average daily gain	/	/	
				Pathological and histopathological lesions	Severe pathological damage	Mild pathological damage	
				Viremia	High viremia	Significantly reduced at some point in time.	
Ingelvac PRRS MLV	28	CHsx1401	21	Clinical symptoms	Severe clinical symptoms	Could not alleviate the clinical signs	[37]
				Days of fever	7 (>40℃)	3 (>40℃)	
				Average daily gain	Significantly lower than negative control group	Significantly lower than negative control group	
				Pathological and histopathological lesions	Moderate histopathological lesions	Could not reduce lung pathological lesions	
				Viremia	High viremia	Partially efficacious in the reduction of viremia	
JXA1-R	28	CHsx1401	21	Clinical symptoms	Severe clinical symptoms	Could not alleviate the clinical signs	
				Days of fever	7 (>40℃)	10 (>40℃)	
				Average daily gain	Significantly lower than negative control group	Significantly lower than negative control group	
				Pathological and histopathological lesions	Moderate histopathological lesions	Could not reduce lung pathological lesions	
				Viremia	High viremia	Partially efficacious in the reduction of viremia	
Ruilanan® (TJM-F92)	28	v2016/ZJ/09-03	15	Clinical symptoms	Severe clinical symptoms	Significantly decreased	[64]
				Days of fever	11 (>41℃)	11 (>41℃)	
				Average daily gain	Significantly lower than negative control group	Significantly lower than negative control group	
				Pathological and histopathological lesions	Typical pathological lesions	Significantly reduced.	
				Viremia	High viremia	No significant reduction	
Ingelvac PRRS® MLV	28	v2016/ZJ/09-03	15	Clinical symptoms	Severe clinical symptoms	Significantly decreased	
				Days of fever	11 (>41℃)	7 (>41℃)	
				Average daily gain	Significantly lower than negative control group	Significantly lower than negative control group	
				Pathological and histopathological lesions	Typical pathological lesions	The lesions were significantly reduced.	

				Viremia	High viremia	Significantly reduced at some point in time.	
Ingelvac PRRS® MLV	28	FJZ03	14	Clinical symptoms	Typical symptoms of PRRS	No clinical symptoms	[44]
				Days of fever	10 (>40℃)	4 (>40℃)	
				Average daily gain	Significantly lower than negative control group	Lower than negative control group but not significantly	
				Pathological and histopathological lesions	Severe pathological lesions	Mild lung lesions	
				Viremia	High viremia	Significantly reduced at some point in time.	
Ingelvac PRRS® MLV	28	FJWQ16	14	Clinical symptoms	Typical symptoms of PRRS	Severe clinical signs	
				Days of fever	Fever days 10 (>40℃)	Fever days 9 (>40℃)	
				Average daily gain	Significantly lower than negative control group	Significantly lower than negative control group	
				Pathological and histopathological lesions	Severe pathological lesions	Mild lung lesions	
				Viremia	High viremia	Significantly reduced at some point in time.	
Ingelvac PRRS® MLV	42	v2016/ZJ/09-03	14	Clinical symptoms	Severe clinical symptoms	Mild cough	[47]
				Days of fever	10~15 (>40℃)	2~12 (>40℃)	
				Average daily gain	Significantly lower than negative control group	Significantly lower than negative control group	
				Pathological and histopathological lesions	Severe pathological damage	Mild to moderate interstitial pneumonia	
				Viremia	High viremia	Significantly reduced	
SD-R	28	SD	21	Clinical symptoms	Severe clinical symptoms	No clinical symptoms	This study
				Days of fever	6~13 (⩾40.5℃)	No fever	
				Average daily gain	Significantly lower than negative control group	No significant difference compared with negative control group	
				Pathological and histopathological lesions	Severe pathological damage	No obvious pathological damage	
				Viremia	High viremia	Significantly reduced	
SD-R	28	HLJWK108-1711	21	Clinical symptoms	Severe clinical symptoms	No clinical symptoms	
				Days of fever	5~10 (⩾40.5℃)	No fever	
				Average daily gain	Significantly lower than negative control group	No significant difference compared with negative control group	
				Pathological and histopathological lesions	Severe pathological damage	No obvious pathological damage	
				Viremia	High viremia	Significantly reduced	
